# Association of IL-4 and IL-10 Polymorphisms With Preterm Birth Susceptibility: A Systematic Review and Meta-Analysis

**DOI:** 10.3389/fimmu.2022.917383

**Published:** 2022-07-04

**Authors:** Xian-Ling Cao, Xuan-You Zhou, Nai-Xin Xu, Song-Chang Chen, Chen-Ming Xu

**Affiliations:** ^1^ Obstetrics and Gynecology Hospital of Fudan University, School of Medicine, Shanghai, China; ^2^ Institute of Reproduction and Development, Fudan University, Shanghai, China; ^3^ International Peace Maternity and Child Health Hospital, School of Medicine, Shanghai Jiao Tong University, Shanghai, China

**Keywords:** preterm birth, genetic polymorphism, IL-4, IL-10, meta-analysis

## Abstract

**Objective:**

Preterm birth (PTB) is a typical inflammatory disease with unclear pathogenesis. The studies investigating the relationship between anti-inflammatory factors IL-4 and IL-10 gene polymorphisms and PTB produced conflicting results. This systematic review and meta-analysis aimed to summarize the effects of IL-4 and IL-10 gene polymorphisms and clarify their possible association with PTB.

**Methods:**

A systematic literature review was conducted using PubMed, Web of Science, and Cochrane library (up to 02 April 2022). The MeSH terms, related entry terms, and other names in “Gene” database were used to find relevant articles. A fixed- or random-effects model was used to calculate the significance of IL-4 and IL-10 gene polymorphisms, depending on study heterogeneity. The odds ratios (OR) and 95% confidence intervals (CIs) were calculated in the allele, recessive, dominant, co-dominant, and over-dominant models. The Eggers publication bias plot was used to graphically represent the publication bias.

**Results:**

Polymorphisms in two interleukins (IL-4-590C/T (rs2243250) = 5 and IL-10-592A/C (rs1800872), -819T/C (rs1800871) and -1082A/G (rs1800896) = 16) were found in 21 articles. Overall, only the over-dominant gene model AA + GG *vs*. AG revealed significant association between IL-10-1082A/G (rs1800896) and PTB (OR [95% CI] = 0.87 [0.76, 0.99], *p* = 0.04). However, in the allele model, recessive model, dominant model, co-dominant model, and over-dominant model, the polymorphisms for IL-4-590C/T (rs2243250), IL-10-592A/C (rs1800872), and IL-10-819T/C (rs1800871) were not found to be associated with the risk of PTB. In gene models, no statistically significant association was found between IL-4-590C/T (rs2243250), IL-10-592A/C (rs1800872), IL-10-819T/C (rs1800871), and IL-10-1082A/G (rs1800896) polymorphisms and PTB in subgroup analyses by racial or control group Hardy-Weinberg Equilibrium (HWE) *p*-value. Eggers’s publication bias plot and heterogeneity test (I^2^<50%, *p* = 0.05) of IL-10-1082A/G (rs1800896) suggested that the funnel asymmetry could be due to publication bias rather than heterogeneity.

**Conclusion:**

The current study suggests that the over-dominant gene model AA + GG *vs*. AG of IL-10-1082A/G (rs1800896) polymorphism may be associated with genetic susceptibility to PTB and may have a protective function against PTB risk. There was unclear association found between IL-4-590C/T (rs2243250), IL-10-592A/C (rs1800872) and IL-10-819T/C (rs1800871) polymorphisms and PTB. Due to the limitations of included studies and the risk of publication bias, additional research is required to confirm our findings.

**Systematic Review Registration:**

https://inplasy.com/inplasy-2022-4-0044, identifier INPLASY202240044.

## Introduction

Preterm birth (PTB) is defined by the World Health Organization (WHO) as babies born alive before 37 weeks of pregnancy (1). According to the new estimates, PTB prevalence in 2014 ranged from 8.7% to 13.4% of all live births, with approximately 15 million preterm babies born each year (2). PTB is the leading cause of death in children under five years globally (3). Furthermore, premature babies are at a higher risk of short- and long-term complications caused by the immaturity of multiple organ systems, such as cerebral palsy, intellectual disabilities, vision and hearing impairments, and impaired cognitive development (4, 5). PTB has become a global public health issue, with mounting evidence indicating a syndrome attributed to various pathological processes (5). Many studies have revealed that genetic variations in pro-inflammatory cytokines such as tumor necrosis factor-α (TNF-α) and interleukin-1α (IL-1α) are associated with an increased risk of PTB (6). However, the relationship between genetic polymorphisms in anti-inflammatory cytokines and PTB risk remains controversial. The transition from a quiescent to a pro-inflammatory environment during pregnancy initiates labor, confirmed by the increasing production of pro-inflammatory cytokines, such as interleukins and TNF-α during labor (7). Cytokines are required during labor to initiate and regulate uterine contractions, cervical ripening, and fetal membrane rupture (7).

In contrast to the up-regulation of PTB-related pro-inflammatory cytokines, anti-inflammatory cytokines such as IL-10 and IL-4 can provide a compensatory protective role during pregnancy by limiting macrophage production of pro-inflammatory cytokines (8, 9). PTB risk is associated with a shift in the cytokine profile toward pro-inflammatory and decreased anti-inflammatory cytokines. However, in women without identifiable infectious agents, high levels of pro-inflammatory cytokines and low levels of anti-inflammatory cytokines may be caused by other factors (e.g., a genetically determined predisposition to up-regulated or down-regulated synthesis of pro-inflammatory and anti-inflammatory cytokines due to gene polymorphisms) (10). Numerous studies have linked cytokines and diseases, particularly some key single nucleotide polymorphisms (SNPs) in these cytokine genes that may interfere with gene expression, potentially impacting disease pathogenesis.

Various cells secrete IL-4 and IL-10. They play an important role in maintaining tissue homeostasis during infection and inflammation by limiting excessive inflammatory responses, enhancing innate immunity, and promoting tissue repair mechanisms (11–13). Therefore, IL-4 and IL-10 may play an important role in PTB, characterized by infection and inflammation (14, 15). *In vitro* experiments revealed that IL-4 and IL-10 could down-regulate lipopolysaccharide (LPS)-mediated inflammatory response in human pregnancy-related tissues (14). Animal studies also found that IL-10 could effectively prevent LPS-induced PTB (15). Therefore, understanding the roles of IL-4 and IL-10 in PTB is beneficial for clinical transformation research and may provide potential targets for PTB treatment.

Many studies have found gene polymorphisms in cytokines like IL-4 and IL-10 may play important roles in PTB. However, the findings are contradictory. As a result of our extensive literature search and preliminary screening, we chose the two most studied anti-inflammatory factors, IL-4 and 10, for further investigation. This systematic review and meta-analysis aimed to determine the relationship between SNPs of anti-inflammatory cytokine gene IL-4 and IL-10 and PTB.

## Methods

### Search Strategy

A systematic literature search was conducted using PubMed, Web of Science, and Cochrane Library, with articles published until 02 April 2022. To ensure a comprehensive literature search, the reference lists of included studies and related reviews were manually searched and screened. There was no language restriction. Two authors (XL and XY) independently searched all databases using the following search terms: (“Polymorphism, Genetic” OR “Polymorphisms, Genetic” OR “Genetic Polymorphism” OR “Genetic Polymorphisms” OR “Gene Polymorphism” OR “Gene Polymorphisms” OR “Polymorphism, Gene” OR “Polymorphisms, Gene” OR “Polymorphism (Genetics)” OR “Polymorphisms (Genetics)” OR “Genetic Variation” OR “Genetic Variations” OR “Variations, Genetic” OR “Variation, Genetic” OR “Diversity, Genetic” OR “Diversities, Genetic” OR “Genetic Diversities” OR “Genetic Diversity”) AND (“Birth, Premature” OR “Births, Premature” OR “Premature Births” OR “Preterm Birth” OR “Birth, Preterm” OR “Births, Preterm” OR “Preterm Births” OR “Premature Birth” OR “Preterm Premature Rupture of the Membranes” OR “PPROM” OR “spontaneous preterm birth” OR “premature labor” OR “Obstetric Labor, Premature” OR “Labor, Premature Obstetric” OR “Preterm Labor” OR “Labor, Preterm” OR “Premature Obstetric Labor” OR “Labor, Premature” OR “Premature Labor” OR “Premature delivery” OR “Preterm delivery”) AND ((“CSIF” OR “TGIF” OR “GVHDS” OR “IL-10” OR “IL10A” OR “Interleukin 10” OR “IL10” OR “CSIF-10” OR “Cytokine Synthesis Inhibitory Factor”) OR (“BSF1” OR “BCGF1” OR “BSF-1” OR “Interleukin 4” OR “Interleukin-4” OR “B-Cell Growth Factor-1” OR “B Cell Growth Factor 1” OR “B-Cell Growth Factor-I” OR “B Cell Growth Factor I” OR “B-Cell Proliferating Factor” OR “B Cell Proliferating Factor” OR “B-Cell Stimulating Factor-1” OR “B Cell Stimulating Factor 1” OR “B-Cell Stimulatory Factor 1” OR “B-Cell Stimulatory Factor-1” OR “BCGF-1” OR “Binetrakin” OR “IL-4” OR “IL4” OR “Mast Cell Growth Factor-2” OR “Mast Cell Growth Factor 2” OR “MCGF-2” OR “B Cell Stimulatory Factor-1” OR “B Cell Stimulatory Factor 1”).

### Inclusion and Exclusion Criteria

The following were the inclusion criteria for our systematic review and meta-analysis: 1) case-control or cohort studies; 2) the case group of patients diagnosed with PTB or preterm premature rupture of membranes (PPROM), and the control group of term birth healthy individuals; 3) investigating the association between IL-4 and IL-10 polymorphisms and PTB or PPROM; 4) sufficient data supporting the genotype distribution were provided for the calculation of odds ratios (ORs) and corresponding 95% confidence intervals (CIs), and 5) full-text articles can be obtained. The following were the exclusion criteria: 1) studies that did not meet any of the inclusion criteria; and 2) studies that contained repeated data.

### Study Selection and Data Extraction

Using Endnote (EndNote 2020), duplicate studies were excluded during data selection. The titles and abstracts of included studies were further screened, and the full text was reviewed using the inclusion and exclusion criteria. The following characteristics were extracted from each eligible literature by two reviewers (XL and XY) independently following the preferred reporting items for systematic reviews and meta-analyses (PRISMA) guidance (16): first author’s surname, publication year, country of the study, age of subjects, the phenotype of case group, single nucleotide polymorphism (SNP) genotyping method, numbers of cases and controls, PTB time of case group, Newcastle-Ottawa Scale (NOS) quality score of each study, Hardy-Weinberg Equilibrium (HWE) *p*-value of controls and the genotype frequencies of IL-4 and IL-10 gene polymorphisms in cases and controls. Discrepancies were resolved through discussion until consensus was reached, and if inconsistencies emerged, an expert (CM) was referred.

### Quality Assessment

The two reviewers (XL and XY) independently assessed the quality of the methods for the included literature using NOS to determine the quality of non-randomized studies in meta-analyses (17). NOS assesses quality utilizing a star rating system; studies with scores ranging from 0 to 9 stars and ≥7 stars were considered high quality. Disputes between the two reviewers were resolved in the same manner described above.

### Assessment of Heterogeneity and Publication Bias

According to Cochrane Q test, statistical heterogeneity was assessed using the standard χ^2^ test (α = 0.1) and I^2^ test. A fixed-effects model (Mantel–Haenszel method) was used to confirm collective effectiveness If *p* ≥ 0.05 and I^2^ ≤ 50% were met. Random-effects models was used if *p* < 0.05 or I^2^ > 50%. Eggers’s publication bias plot was used to represent the publication bias graphically. Trim-and-fill method was used to adjust the publication bias. Besides, publication bias (*p* < 0.05) was carefully discussed in our discussion section and listed as one of the limitations of our meta-analysis.

### Statistical Analysis

The meta-analysis was performed using Stata (version 17.0) software. ORs and 95% CIs were calculated to assess the association between IL-4-590C/T (rs2243250), IL-10-592A/C (rs1800872) (referred as T/G in “dpSNP” database), IL-10-819T/C (rs1800871) (referred as A/G in “dpSNP” database), and IL-10-1082A/G (rs1800896) (referred as T/C in “dpSNP” database) polymorphisms and PTB in the allele model T-allele *vs*. C-allele, C-allele *vs*. A-allele, C-allele *vs*. T-allele and G-allele *vs*. A-allele, recessive model TT *vs*. CT + CC, CC *vs*. AA + AC, CC *vs*. TT + TC and GG *vs*. AA + AG, dominant model CT+TT *vs*. CC, AC + CC *vs*. AA, TC + CC *vs*. TT and AG + GG *vs*. AA, co-dominant model TT *vs*. CC and CT *vs*. CC, CC *vs*. AA and AC *vs*. AA, CC *vs*. TT and TC *vs*. TT, AG *vs*. AA and GG *vs*. AA and over-dominant model CC + TT *vs*. CT, AA + CC *vs*. AC, TT + CC *vs*. TC and AA + GG *vs*. AG respectively. According to HWE, in the case of a pair of alleles, the relationship between gene p (dominant) and gene q (recessive) gene frequency is as follows: *p* + *q* = 1, *p2* + 2*pq* + *q2*. If only genotype or allele frequency information was provided in the included study, we used this formula to calculate the number of specific gene models. Subgroup analysis was used to investigate the potential sources of heterogeneity by racial or by HWE *p*-value of controls. The goodness-of-fit χ2 test assessed the control group’s HWE deviation. We performed sensitivity analyses to determine the robustness of our findings. For sensitivity analysis, a leave-one-out meta-analysis was used. A *p*-value < 0.05 was deemed statistically significant.

### Trial Sequential Analysis

Trial sequential analysis (TSA) was performed by using the TSA-Trial Sequential Analysis Viewer (version 0.9.5.10 β, Copenhagen Trial Unit, Copenhagen, Denmark) (18). The required information size and TSA monitoring boundaries were then generated automatically.

### Expression Quantitative Trait Loci Assessment for SNPs

We searched for expression quantitative trait Loci (eQTL) for the SNPs, using one publicly available database (QTLbase: http://www.mulinlab.org/qtlbase/index.html). Results were presented as nominal *p* values for each SNP.

## Results

### Study Selection

**Figure 1:**
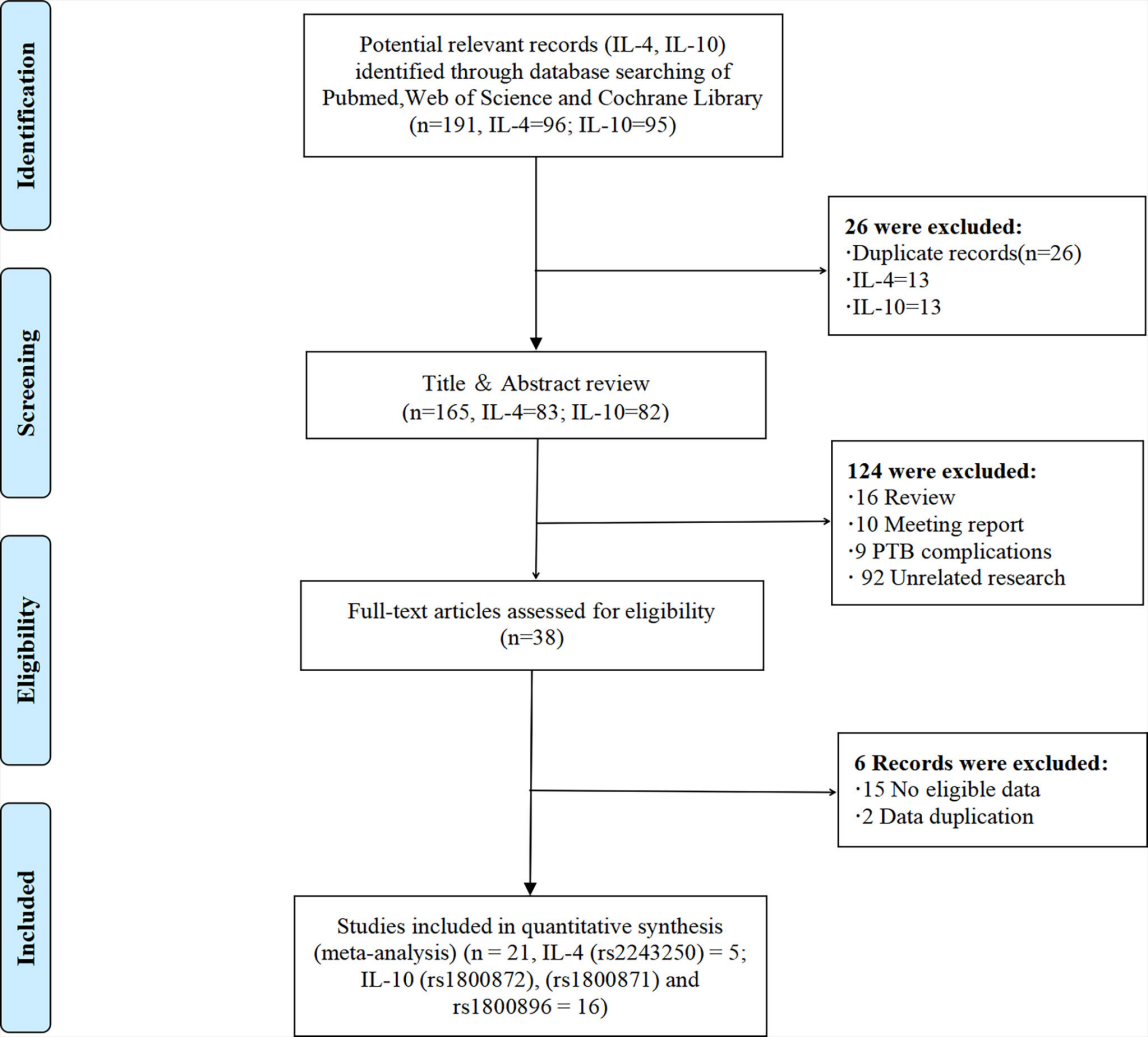
The PRISMA flow diagram. PRISMA, Preferred Reporting Items for Systematic Reviews and Meta-Analyses.

According to PRISMA flow diagram, a systematic literature search of PubMed, Web of Science, and Cochrane library yielded 191 studies, including 26 duplicate records (**Figure 1**, and **Table S1**). We excluded 124 studies after reviewing the titles and abstracts of 165 non-duplicate articles, for the following reasons: 1) studies were review articles (n = 16); 2) studies were meeting reports (n = 10); 3) studies were about PTB complications (n = 9); and 4) other unrelated studies (n = 92). Of the remaining 38 studies, 15 were excluded because they lacked eligible data, and one study was excluded because its data was duplicated (19). Finally, 21 eligible studies (IL-4 = 5, IL-10 = 16, contains four repetitive studies) with a total sample size ranging from 29 to 559 cases were included in this study. These studies included PTB or PPROM cases as well as healthy controls. Using these studies, we investigated the relationship between alleles and genotypes of the IL-4-590C/T (rs2243250), IL-10-592A/C (rs1800872), IL-10-819T/C (rs1800871), and IL-10-1082A/G (rs1800896) genetic polymorphisms and the risk of PTB.

### Study Characteristics

**Table 1** summarizes the key characteristics of included studies in our systematic review meta-analysis. From 2004 to 2020, these studies included 538 cases and 1123 controls for IL-4-590C/T (rs2243250), 787 cases and 1756 controls for IL-10-592A/C (rs1800872), 1225 cases and 1769 controls for IL-10-819T/C (rs1800871) and 1612 cases and 3386 controls for IL-10-1082A/G (rs1800896), respectively. Because not all genotypes were identified, the number of genotypes was somewhat inconsistent with the number of cases and controls. Most case groups were PTB patients, with some studies including PPROM patients. Most studies had NOS scores greater than or equal to 7, indicating high quality.

**Table 1:** Main characteristics of included studies.

IL-4-590C/T (rs2243250)																
Author	Year	Cases/Controls	Racial	Age (mean ± SD) or [range]		SNP GM	Cases			Controls			HWE(*P*) control	Phenotype	PTB time	NOS score
				Cases	Controls		TT	CT	CC	TT	CT	CC				
Lyubomirskaya et al	2020	50/50	Ukrainian	29.60 ± 6.30		TaqMan-SNP	2	11	37	3	19	28	0.92	PPROM	26-34W	8
Heinzmann et al	2009	121/270	German	NM	NM	PCR-RFLP	2	25	94	7	74	189	0.95	PTB	<32W	6
Kalish et al	2004	29/44	American	NM	NM	PCR-SSP	6	9	14	1	14	29	0.65	PTB	<37W	7
Annells et al	2004	202/185	Australian	29.33[15-44]	30[18-43]	PCR-SSP	4	36	162	2	46	137	0.39	PTB	20-35W	9
Engel et al	2005	136/574	American	26.60 ± 6.40	26.60 ± 6.10	TaqMan-SNP	5	44	87	10	130	434	0.94	PTB	24-29W	7
**IL-10-592A/C (rs1800872)**																
Author	Year	Cases/ Controls	Racial	Age (mean ± SD) or [range]		SNP GM	Cases			Controls			HWE(*P*) control	Phenotype	PTB time	NOS score
				Cases	Controls		CC	AC	AA	CC	AC	AA
Lyubomirskaya et al	2020	50/50	Ukrainian	29.60 ± 6.30		TaqMan-SNP	25	18	7	2	37	11	0.00	PPROM	26-34W	8
Han et al	2020	115/147	Korean	30.52 ± 4.60	30.47 ± 4.89	PCR-RFLP	14	58	43	20	56	71	0.10	PTB	24-37W	9
Pandey et al	2018	559/559	Indian	25.85 ± 4.17	25.67 ± 3.72	ARMS-PCR	334	147	78	355	145	59	0.00	PTB	<37W	8
Ramos et al	2016	201/201	Brazilian	22.80 ± 6.30 26.20 ± 6.20	23.90 ± 6.10	TaqMan-SNP	69	53	10	79	93	27	0.96	PTB/ PPROM	<37W	7
Menon et al	2010	83/40	American	25.32 ± 5.53 27.33 ± 6.30	25.22 ± 5.29 27.33 ± 6.30	NM	45	33	5	26	10	4	0.07	PTB	22-36W	6
Engel et al	2005	136/574	American	26.60 ± 6.40	26.60 ± 6.10	TaqMan-SNP	69	55	12	256	247	71	0.34	PTB	24-29W	7
Annells et al	2004	202/185	Australian	29.33[15-44]	30[18-43]	PCR-SSP	123	69	10	112	64	9	0.97	PTB	20-35W	9
**IL-10-819T/C(rs1800871)**																
Author	Year	Cases/ Controls	Racial	Age (mean ± SD) or [range]		SNP GM	Cases			Controls			HWE(*P*) control	Phenotype	PTB time	NOS score
				Cases	Controls		CC	TC	TT	CC	TC	TT
Lyubomirskaya et al	2020	50/50	Ukrainian	NM	NM	TaqMan-SNP	11	2	37	4	7	39	0.00	PPROM	26-34W	8
Pandey et al	2018	559/559	Indian	25.85 ± 4.17	25.67 ± 3.72	ARMS-PCR	285	190	84	304	180	76	0.00	PTB	<37W	8
Ramos et al	2016	201/201	Brazilian	22.80 ± 6.30 26.20 ± 6.20	23.90 ± 6.10	TaqMan-SNP	64	56	12	78	93	28	0.97	PTB/ PPROM	<37W	7
Nuk et al	2012	77/200	Austrian	31[26-35]	29[24-34]	PCR-SSP	36	31	6	109	73	15	0.57	PTB	29-30W	7
Engel et al	2005	136/574	American	26.60 ± 6.40	26.60 ± 6.10	TaqMan-SNP	69	55	12	256	247	71	0.34	PTB	24-29W	7
Annells et al	2004	202/185	Australian	29.33[15-44]	30[18-43]	PCR-SSP	123	69	10	112	64	9	0.97	PTB	20-35W	9
**IL-10-1082A/G (rs1800896)**																
Author	Year	Cases/ Controls	Racial	Age (mean ± SD) or [range]		SNP GM	Cases			Controls			HWE(*P*) control	Phenotype	PTB time	NOS score
				Cases	Controls		GG	AG	AA	GG	AG	AA
Pandey et al	2018	559/559	Indian	25.85 ± 4.17	25.67 ± 3.72	ARMS-PCR	190	291	78	246	257	56	0.35	PTB	<37W	8
Nuk et al	2012	77/200	Austrian	31[26-35]	29[24-34]	PCR-SSP	11	43	23	30	90	60	0.70	PTB	29-30W	7
Menon et al	2010	83/40	American	25.32 ± 5.53 27.33 ± 6.30	25.22 ± 5.29 27.33 ± 6.30	NM	13	53	17	9	19	12	0.78	PTB	22-36W	6
Moura et al	2009	192/198	Brazilian	24.10 ± 6.20	23.60 ± 6.60	PCR-SSP	25	100	67	24	93	81	0.73	PTB	24-36+6W	7
Stonek et al	2008	86/1362	Austrian	23.70 ± 4.70		PCR-RFLP	14	52	20	237	669	456	0.76	PTB	<37W	7
Speer et al	2006	80/80	American	26.90 ± 6.60	26.40 ± 6.70	PCR-SSP	16	41	19	17	39	23	0.95	PTB	<37W	8
Mattar et al	2006	139/119	Brazilian	35[18-45]	NM	PCR-SSP	15	68	51	15	57	47	0.72	PTB	<37W	7
Kerk et al	2006	29/25	German	32[22-44]	32[20-36]	PCR-RFLP	6	19	4	5	11	9	0.63	PTB	<29W	8
Engel et al	2005	136/574	American	26.60 ± 6.40	26.60 ± 6.10	TaqMan-SNP	69	55	12	256	247	71	0.34	PTB	24-29W	7
Kalish et al	2004	29/44	American	34[20-49]		PCR-SSP	4	14	9	8	19	14	0.73	PTB	24-37W	7
Annells et al	2004	202/185	Australian	29.33[15-44]	30[18-43]	PCR-SSP	61	85	56	40	101	44	0.21	PTB	20-35W	9

SD, standard deviation; HWE, Hardy-Weinberg Equilibrium;NOS, Newcastle-Ottawa Scale; PPROM, preterm premature rupture of membranes; GM, genotype method; W, week; PCR-RFLP, polymerase chain reaction restriction fragment length polymorphism; NM, not mentioned; PTB, preterm birth; VNTR, variable number of tandem repeats; PCR-SSP, polymerase chain reaction and sequence specific primers; ARMS-PCR, multiplex amplification refractory mutation system; SNP, single nucleotide polymorphism.

### Results of Meta-Analysis

Five studies in our review investigated the association between IL-4-590C/T (rs2243250) polymorphism and PTB. No significant association was identified between the overall risk of PTB and SNP under different genetic models (**Table 2** and **Figure S1**). A racial subgroup analysis revealed no significant association between PTB and IL-4-590C/T (rs2243250) polymorphism under different genetic models (**Figure S2**).

**Table 2:** Main results of the total and subgroup analysis.

IL-4-590C/T (rs2243250)																					
Study groups	T-allele vs. C-allele			CT+TT vs. CC					TT vs. CT+CC				TT vs. CC			CT vs. CC			CC+TT vs. CT		
	OR(95% CI)		*P* value	OR(95% CI)				*P* value	OR(95% CI)			*P* value	OR(95% CI)		*P* value	OR(95% CI)		*P* value	OR(95% CI)		*P* value
Overall	1.03[0.61,1.75]		0.91	0.94[0.55,1.62]				0.83	1.73[0.91,3.28]			0.09	1.68[0.88,2.20]		0.12	0.87[0.53,1.42]		0.57	1.20[0.75,1.89]		0.45
**Subgroup analysis** By racial																					
Ukrainian	0.53[0.26,1.08]		–	0.45[0.19,1.04]				–	0.65[0.10,4.09]			–	0.50[0.08,3.23]		–	0.44[0.18,1.17]		–	2.17[0.90,5.24]		–
German	0.70[0.45,1.10]		–	0.67[0.41,1.11]				–	0.63[-.13,3.09]			–	0.57[0.12,2.82]		–	0.68[0.41,1.14]		–	1.45[0.87,2.43]		–
American	1.79[1.28,2.50]		0.30	1.79[1.24,2.59]				0.75	3.47[1.41,8.57]			0.17	3.96[1.58,9.88]		0.19	1.64[1.11,2.40]		0.68	0.66[0.45,0.96]		0.34
Australian	0.78[0.51,1.21]		–	0.70[0.44,1.14]				–	1.85[0.33,10.21]			–	1.69[0.31,9.38]		–	0.66[0.40,1.08]		–	1.53[0.93,2.49]		–
**IL-10-592A/C (rs1800872)**																					
Study groups	C-allele *vs*. A-allele			AC+CC *vs*. AA					CC *vs*. AA+AC				CC *vs*. AA			AC *vs*. AA			AA+CC *vs*. AC		
	OR(95% CI)		*P* value	OR(95% CI)				*P* value	OR(95% CI)			*P* value	OR(95% CI)		*P* value	OR(95% CI)		*P* value	OR(95% CI)		*P* value
Overall	1.19[0.89,1.60]		0.24	1.13[0.90,1.42]				0.29	1.31[0.74,2.31]			0.36	1.51[0.86,2.64]		0.15	1.12[0.87,1.44]		0.36	1.09[0.70,1.69]		0.71
**Subgroup analysis** By racial																					
Ukrainian	3.06[1.11,5.46]		–	1.73[0.61,4.91]				–	24[5.25,109.65]			–	19.64[3.50,114.14]		–	0.76[0.25,2.30]		–	5.06[2.15,11.91]		–
Korean	1.09[0.75,1.58]		–	1.56[0.95,2.57]				–	0.88[0.42,1.83]			–	1.16[0.53,2.52]		–	1.71[1.01,2.90]		–	0.60[0.37,0.99]		–
American	1.13[0.79,1.60]		0.25	1.50[0.84,2.69]				0.82	0.99[0.51,1.91]			0.11	1.55[0.85,2.84]		0.86	1.46[0.78,2.74]		0.41	0.82[0.39,1.74]		0.09
Australian	0.94[0.67,1.32]		–	0.98[0.39,2.47]				–	1.01[0.67,1.53]			–	0.99[0.39,2.52]		–	0.97[0.37,2.54]		–	1.02[0.67,1.55]		–
Indian	0.83[0.68,1.00]		–	0.73[0.51,1.04]				–	0.85[0.67,1.09]			–	0.71[0.49,1.03]		–	0.77[0.51,1.15]		–	0.98[0.75,1.28]		–
Brazilian	1.53[1.09,2.15]		–	1.92[0.89,4.10]				–	1.66[1.07,2.59]			–	2.36[1.07,5.22]		–	1.54[0.69,3.42]		–	1.31[0.84,2.04]		–
By HWE *p* value																					
≤0.1	1.20[0.68,2.13]		0.00	1.01[0.77,1.33]				0.05	1.64[0.36,7.45]			0.00	1.83[0.50,6.63]		0.00	1.05[0.78,1.42]		0.06	1.08[0.41,2.84]		0.00
>0.1	1.22[0.94,1.59]		0.13	1.47[0.96,2.26]				0.55	1.28[0.99,1.66]			0.28	1.51[0.86,2.64]		0.86	1.30[0.82,2.04]		0.77	1.13[0.89,1.44]		0.72
**IL-10-819T/C (rs1800871)**																					
Study groups	C-allele *vs*. T-allele			TC+CC *vs*. TT					CC *vs*. TT+TC				CC *vs*. TT			TC *vs*. TT			TT+CC *vs*. TC		
	OR(95% CI)		*P* value	OR(95% CI)				*P* value	OR(95% CI)			*P* value	OR(95% CI)		*P* value	OR(95% CI)		*P* value	OR(95% CI)		*P* value
Overall	1.04[0.92,1.17]		0.56	1.07[0.84,1.36]				0.59	1.04[0.89,1.21]			0.66	1.11[0.86,1.43]		0.41	1.03[0.79,1.34]		0.84	1.01[0.86,1.19]		0.92
**Subgroup analysis** By HWE *p* value																					
<0.05	0.93[0.78,1.11]		0.07	0.92[0.67,1.27]				0.50	0.92[0.74,1.16]			0.04	0.94[0.67,1.31]		0.06	0.89[0.62,1.28]		0.18	0.96[0.75,1.23]		0.08
>0.05	1.14[0.97,1.35]		0.23	1.32[0.90.1.94]				0.72	1.14[0.92,1.42]			0.29	1.40[0.94,2.09]		0.50	1.23[0.82,1.85]		0.25	1.05[0.84,1.30]		0.71
**IL-10-1082A/G (rs1800896)**																					
Study groups		G-allele *vs*. A-allele					AG+GG *vs*. AA				GG *vs*. AA+AG				AG *vs*. AA			AA+GG *vs*. AG			
		OR(95% CI)			*P* value		OR(95% CI)			*P* value	OR(95% CI)			*P* value	OR(95% CI)		*P* value	OR(95% CI)		*P* value	
Overall		0.99[0.90,1.09]			0.82		1.10[0.93,1.29]			0.27	0.90[0.77,1.05]			0.16	1.13[0.95,1.34]		0.16	0.87[0.76,0.99]		0.04	
**Subgroup analysis** By racial																					
German		1.59[0.74,3.40]			–	3.52[0.93,13.35]				–	1.04[0.28,3.04]			–	3.89[0.97,15.64]			0.41[0.14,1.24]		–	
American		1.15[0.93,1.42]			0.83	1.36[0.92,2.00]				0.90	1.10[0.81,1.50]			0.50	1.39[0.92,2.08]		0.86	0.91[0.69,1.22]		0.34	
Australian		1.10[0.83,1.45]			–	0.81[0.51,1.29]				–	1.57[0.99,2.49]			–	0.66[0.41,1.08]		–	1.66[1.11,2.47]		–	
Austrian		1.13[0.89,1.43]			0.52	1.44[0.98,2.10]				0.38	0.89[0.56,1.41]			0.83	1.52[1.02,2.26]		0.39	0.69[0.49,0.97]		0.53	
Brazilian		1.09[0.87,1.37]			0.53	1.20[0.87,1.64]				0.56	1.00[0.62,1.60]			0.66	1.22[0.87,1.70]		0.63	0.84[0.62,1.15]		0.78	
Indian		0.74[0.62,0.88]			–	0.69[0.48,0.99]				–	0.66[0.51,0.83]			–	0.81[0.55,1.19]		–	0.78[0.62,0.99]		–	

CI, confidence interval; OR, odds ratio; HWE, Hardy-Weinberg Equilibrium.

Seven studies evaluated the relationship between IL-10-592A/C (rs1800872) polymorphism and PTB. No significant association was found between the overall risk of PTB and rs1800872 under different genetic factors models (**Table 2** and **Figure S3**). Subgroup analysis by racial and HWE *p*-value of controls also indicated no significant association between PTB and the IL-10-592A/C (rs1800872) polymorphism under different genetic models (**Figures S4** and **S5**).

Six studies were included to investigate the relationship between IL-10-819T/C (rs1800871) polymorphism and PTB, and no significant association was found between the overall risk of PTB and rs1800871 under different genetic models (**Table 2**, and **Figure S6**). Subgroup analysis by HWE *p-*value of controls also revealed no significant association between PTB risk and the IL-10-819T/C (rs1800871) polymorphism under different genetic models (**Figure S7**).

**Figure 2:**
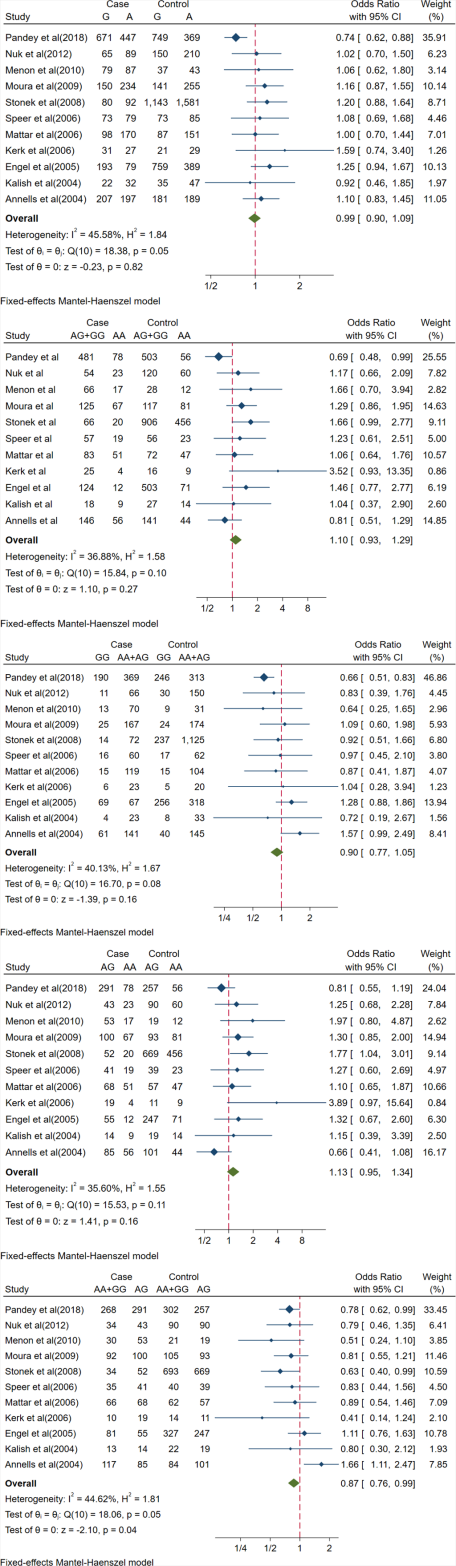
Forest plots of IL-10-1082A/G(rs1800896) polymorphism and PTB. The allele model (G vs A), recessive model (GG vs. AA+AG), dominant model (AG+GG vs AA), co-dominant model (AG vs. AA) and over-dominant model (AA+GG vs. AG). The count for genotypes, weight, OR, 95% confidence interval for each study. The fixed effect and random effect models were respectively utilized according to heterogeneity.

Eleven studies were included to investigate the link between the IL-10-1082A/G (rs1800896) polymorphism and PTB risk. The over-dominant gene model AA + GG *vs*. AG revealed a significant association between IL-10-1082A/G (rs1800896) and PTB (OR [95% CI] = 0.87[0.76, 0.99], *p* = 0.04) (**Table 2** and **Figure 2**). Under the over-dominant gene model, this polymorphism had a protective effect against PTB susceptibility. In the Austrian, American, Brazilian, Brazilian, and German subgroups, there was no significant association between PTB risk and rs1800896 under different genetic models (**Figure S8**). However, a potential significant association between IL-10-1082A/G (rs1800896) and PTB was found from the allele model G-allele *vs*. A-allele, recessive model GG *vs*. AA + AG, dominant model AG + GG *vs*. AA, and over-dominant model AA + GG *vs*. AG (**Figure S8**) in Indian. However, it should be noted that the sample size is small, and only one Indian study is included.

**Figure 3:**
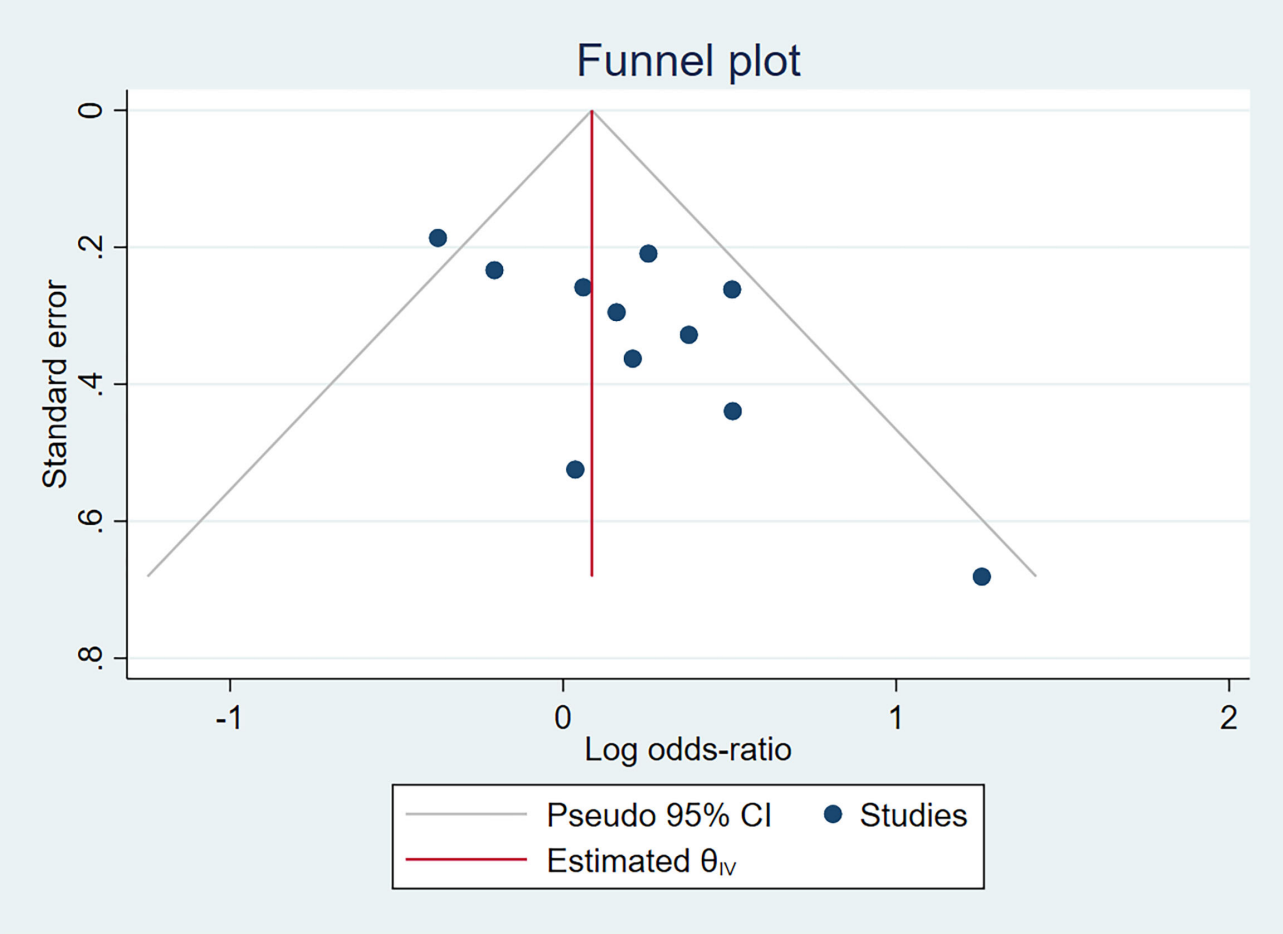
Funnel plots of publication bias of IL-10-1082A/G(rs1800896) polymorphism.

In terms of IL-10-1082A/G (rs1800896) publication bias, there was a relatively obvious asymmetry of the funnel plot (**Figure 3**), indicating some degree of publication bias. It is unlikely to be the publication bias caused by heterogeneity because of low heterogeneity, as displayed in **Figure 2**. Funnel plot assessment of IL-4-590C/T (rs2243250), IL-10-592A/C (rs1800872), and IL-10-819T/C (rs1800871) by visual inspection was symmetrical (**Figure S9**), indicating no significant publication bias.

**Figure 4:**
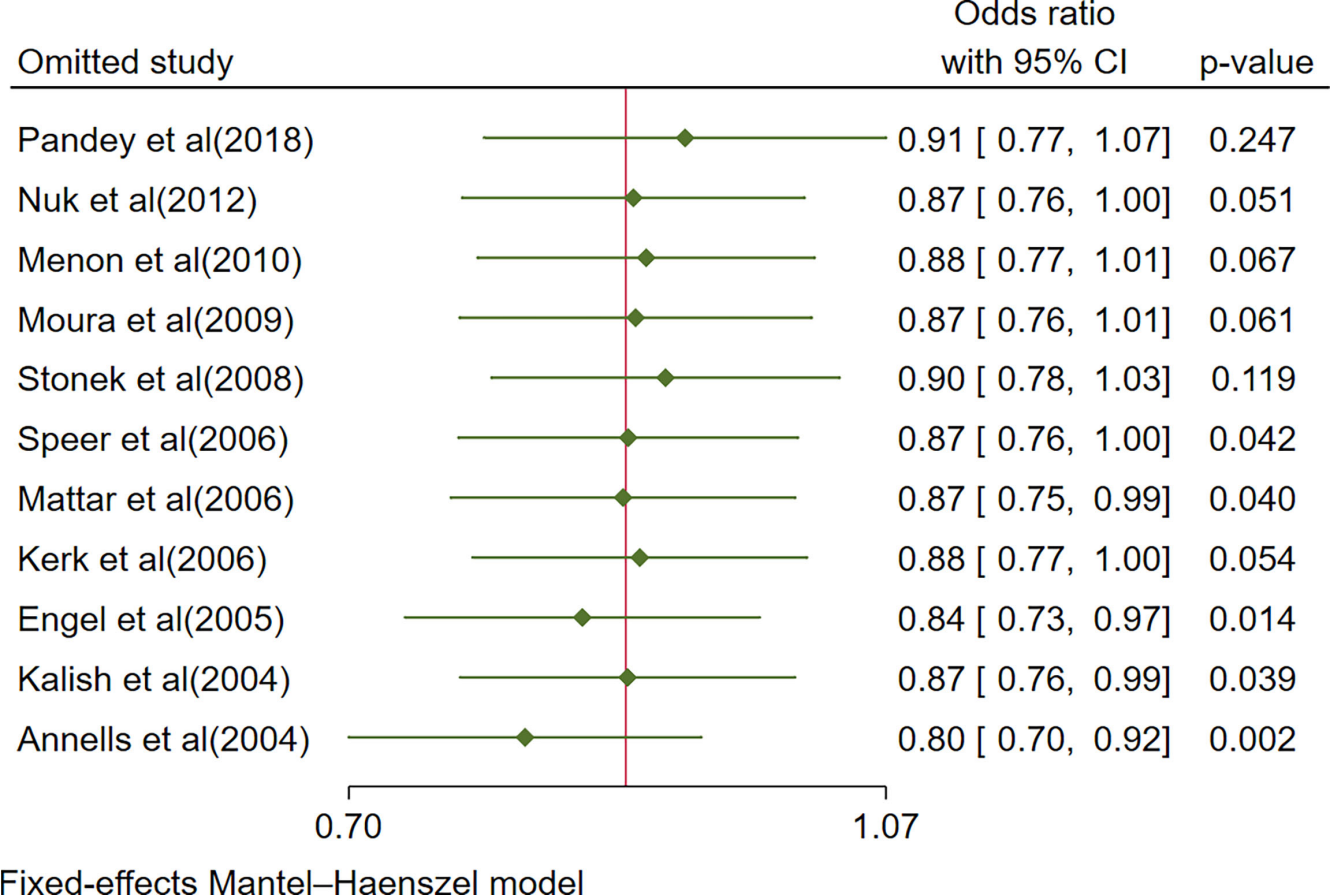
Sensitivity analysis: Leave-one-out meta-analysis of IL-10-1082A/G(rs1800896) polymorphism and PTB (AA+GG vs. AG).

A sensitivity analysis of the included studies was performed using leave-one-out forest plots according to a random- or fixed-effects model to investigate the sensitivity of our results. The sequential exclusion of studies had no significant impact on all of the above findings, indicating their robustness (**Figures 4** and **S10**).

### TSA Analyses

**Figure 5:**
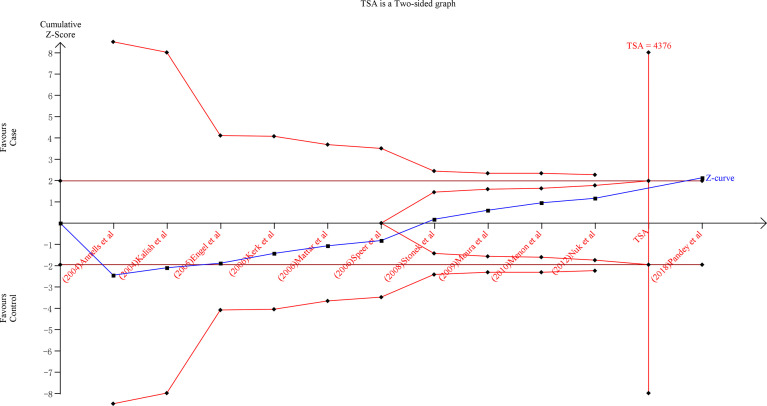
TSA between IL-10-1082A/G(rs1800896) polymorphism and PTB risk (AA+GG vs. AG).

To improve the robustness of our findings, we performed TSA on the over-dominant gene model (AA + GG *vs*. AG) of IL-10-1082A/G (rs1800896), the allele model T-allele *vs*. C-allele of IL-4-590C/T (rs2243250), C-allele *vs*. A-allele of IL-10-592A/C(rs1800872) and C-allele *vs*. T-allele of IL-10-819T/C (rs1800871) polymorphisms (**Figures 5** and **S11**). TSA was performed in all gene models of each polymorphism, and similar results were found, so the above results were used as an example. The findings indicate that the cumulative Z-curve for the over-dominant gene model (AA + GG *vs*. AG) of IL-10-1082A/G (rs1800896) polymorphism crossed the trial sequential monitoring boundary (type I error 5%, Z score = 1.96) before reaching the required information size (TSA = 4376) (**Figure 5**). Similar patterns were observed in the allele model of IL-10-592A/C (rs1800872) and IL-10-819T/C (rs1800871) polymorphisms (TSA = 5142 and 34107, respectively) (**Figure S11**). However, the cumulative Z-curve for the allele model of IL-4-590C/T (rs2243250) polymorphism did not cross the trial sequential monitoring boundary (type I error 5%, Z score = 1.96) and did not reach the required information size (TSA = 10223) (**Figure S11**). As a result, the cumulative evidence was sufficient to support the conclusions for IL-10-1082A/G (rs1800896), IL-10-592A/C (rs1800872), and IL-10-819T/C (rs1800871) polymorphisms and PTB in the corresponding gene model. Furthermore, more research is required to confirm the true relationship between IL-4-590C/T (rs2243250) polymorphism and PTB in different gene models.

### eQTLs for SNPs

To determine whether the above SNPs show evidence as eQTLs in blood, we examined the “QTLbase” for eQTLs. The effective allele and *p* value for eQTLs of both four SNPs were statistically significant (*p* < 10^−8^) (**Table S2**).

## Discussion

In this systematic review and meta-analysis, the over-dominant gene model AA+GG *vs*. AG revealed a significant association between IL-10-1082A/G (rs1800896) and PTB. Furthermore, due to an insufficient number of studies for meta-analysis, many other IL-4 and IL-10 gene polymorphisms were excluded from this study.

Human parturition is an inflammatory process, and when the inflammatory balance is disrupted, the formation of an inflammation cascade can lead to the occurrence of PTB (20). Anti-inflammatory cytokines are essential for pregnancy maintenance (21). Moreover, the anti-inflammatory cytokines IL-4 and IL-10 inhibit the production of pro-inflammatory cytokines, and decreased production of IL-4 and IL-10 may increase susceptibility to infections and lead to PTB (22). When comparing preterm and term placentas, the researchers discovered that term placentas had higher IL-4 and IL-10 (23). Furthermore, studies have indicated that the inflammatory response of PTB is genetically controlled (24). As a result, SNPs in IL-4 and IL-10 may result in lower anti-inflammatory cytokines, shifting the cytokine balance toward inflammation. Therefore, SNPs in IL-4 and IL-10 had been extensively studied as potential candidates for predicting PTB risk (10, 25).

In multifetal pregnancies, IL-4 C > T substitution at position -590 had been associated with increased production of IL-4 and susceptibility to PTB (26). Similarly, Heinzmann et al. and Belousova et al. found an association between IL-4-590C/T (rs2243250) gene polymorphism and the risk of PTB (8, 27). Lyubomirskaya et al., on the other hand, found no significant associations between IL-4 SNPs and risk or protective effect for PPROM in the population of Zaporizhzhia region of Ukraine (28). This meta-analysis found no significant association between IL-4-590C/T (rs2243250) gene polymorphism and PTB. However, IL-4 590 T allele frequency was disproportionately high in certain ethnic groups (29). Our race-based subgroup analysis also found no significant link between the IL-4-590C/T (rs2243250) gene polymorphism and PTB. This could be due to the small number of included studies and the considerable heterogeneity among studies, which requires to be confirmed by further research.

Another well-known anti-inflammatory cytokine that can suppress pro-inflammatory cytokines is IL-10 (10). Genetic variations in IL-10 may affect gene expression and thus its levels, resulting in an inflammatory imbalance in pregnant women and PTB (30). Polymorphisms in IL-10-592A/C (rs1800872), IL-10-819T/C (rs1800871) and IL-10-1082A/G (rs1800896) are common SNPs in PTB studies (9). However, studies on IL-10 gene polymorphisms and PTB have yielded contradictory results (9, 31–34). Menon et al. found that the difference in the interaction of IL-10 SNPs and PTB may have a genetic basis (35). Simultaneously, Moura et al., Stonek et al., and Mattar et al. suggested that IL-10-1082A/G (rs1800896) polymorphism was not a genetic marker for identifying women at increased risk of PTB (25, 36, 37). However, our findings demonstrate that IL-10-1082A/G (rs1800896) over-dominant gene model AA+GG *vs*. AG has a significant protective effect against PTB. Similarly, Kerk et al. revealed that IL-10-1082A/G (rs1800896) might play a role in severe PTB that lasts less than 29 weeks (38). No significant association was found between other gene models of IL-10-592A/C (rs1800872) and IL-10-819T/C (rs1800871) and PTB. Furthermore, no racial differences were found in the subgroup analysis.

Eskdale et al. found that the ability to secrete IL-10 varies in human according to the genetic composition of the IL-10 locus (39). Similarly, Suárez et al. found that IL-10 (mRNA and serum protein) levels showed remarkable inter-individual variations, which are genetically controlled by polymorphic variants at the gene promoter (40). There was a trend of higher IL-10 levels among individuals homozygous for the 1082 G genotype, but no difference in the expression of IL-10 between AG and AA genotype groups (40). The study also found that the concentration of IL-10 transcripts was quite dispersed (interquartile range = 13.85 and 18.87) in individuals with -1082 GG and GA genotypes, indicating that other genetic or environmental factors were also involved (40). In addition, *in vitro* and animal studies demonstrated that IL-10 could improve the outcome of LPS-induced PTB model (14, 15). Clinical studies revealed that lower IL-10 levels were significantly associated with PTB and had the potential to be used as a biomarker for predicting PTB (41). The eQTL assessment showed that the four SNPs (rs2243250, rs1800872, rs1800871 and rs1800896) in this study were significantly correlated with the expression of the corresponding genes (IL-4 and IL-10), which also proved that the protective effect of the over-dominant gene model AA+GG vs AG of IL-10-1082A/G (rs1800896) polymorphism on PTB may be due to the increase of IL-10 expression in blood. Overall, these findings suggested that the protective effect of AA+GG vs. AG of IL-10-1082A/G (rs1800896) genotype on PTB may be due to an increase in IL-10 levels. Furthermore, the difference between -1082 AG and AA genotype could be due to other unknown genetic or environmental factors.

Our study has some limitations as well. First, the number of studies involving IL-4-590C/T (rs2243250), IL-10-592A/C (rs1800872) and IL-10-819T/C (rs1800871) is small. Second, we only performed subgroup analysis on different countries but did not analyze among different races in countries, which may have obscured the correlation caused by race differences. Third, there was a risk of publication bias for IL-10-1082A/G (rs1800896), and data for some genotypes were obtained by calculating, which may differ from the original data. Finally, the absence of publication bias indicated a significant heterogeneity in the included studies of IL-4-590C/T (rs2243250), IL-10-592A/C (rs1800872), and IL-10-819T/C (rs1800871).

In conclusion, our findings suggest that the over-dominant gene model AA + GG *vs*. AG of IL-10-1082A/G (rs1800896) polymorphism may be associated with genetic susceptibility to PTB and may have a protective role. On the other hand, IL-4-590C/T (rs2243250), IL-10-592A/C (rs1800872) and IL-10-819T/C (rs1800871) polymorphisms are unrelated to PTB. Due to the limitations of included studies and the risk of publication bias, future studies in larger populations are required to confirm our findings.

## Data Availability Statement

The original contributions presented in the study are included in the article/**Supplementary Material**. Further inquiries can be directed to the corresponding author.

## Author Contributions

X-LC conceived the idea for this meta-analysis. All authors (X-LC, X-YZ, N-XX, S-CC, C-MX) developed the methodology for the meta-analysis. The manuscript was drafted by X-LC, and revised by X-YZ, N-XX, S-CC, and C-MX. All authors contributed to the article and approved the submitted version.

## Funding

This project is supported by the National Natural Science Foundation of China (NO.82171677).

## Conflict of Interest

The authors declare that the research was conducted in the absence of any commercial or financial relationships that could be construed as a potential conflict of interest.

## Publisher’s Note

All claims expressed in this article are solely those of the authors and do not necessarily represent those of their affiliated organizations, or those of the publisher, the editors and the reviewers. Any product that may be evaluated in this article, or claim that may be made by its manufacturer, is not guaranteed or endorsed by the publisher.
