# Supplementary figures and images for: Association of IL-4 and IL-10 Polymorphisms With Preterm Birth Susceptibility: A Systematic Review and Meta-Analysis
Source: Front Immunol. 2022 Jul 4;13:917383. doi: 10.3389/fimmu.2022.917383 (PMC9289468; doi:10.3389/fimmu.2022.917383)

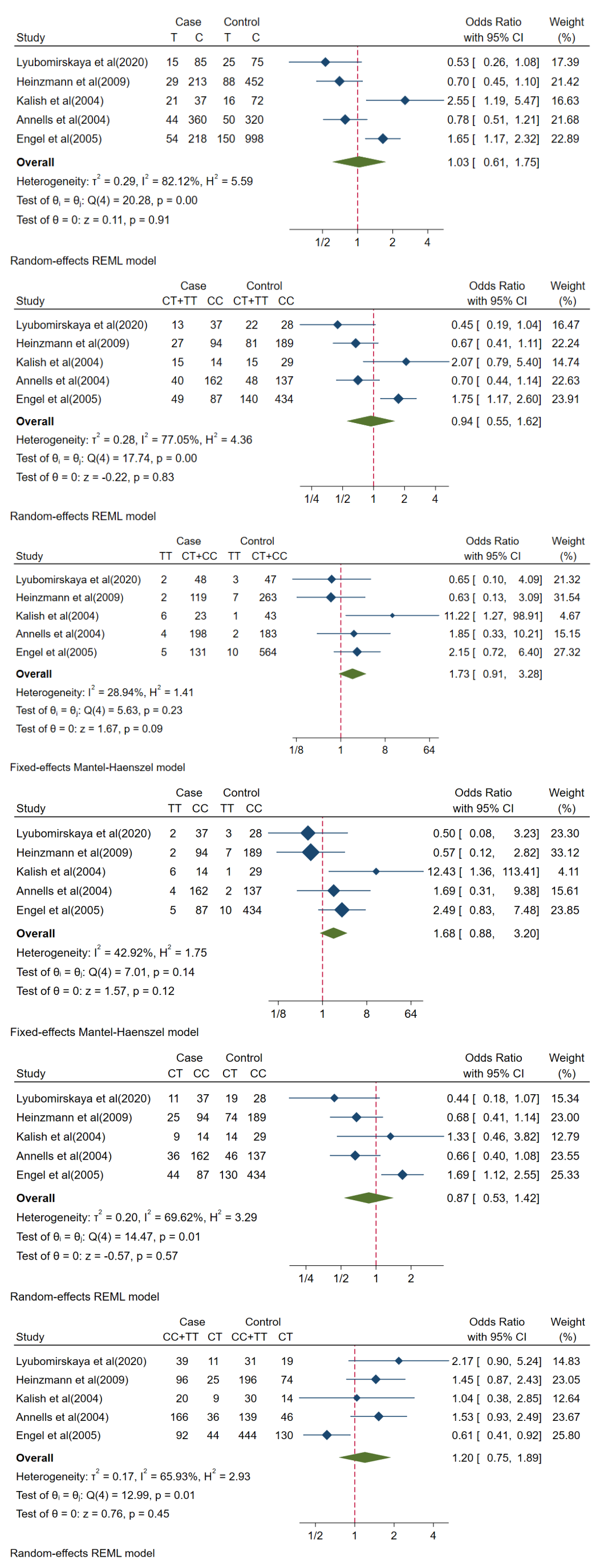

Supplement: Supplementary file 1 [file Image_1.tif]

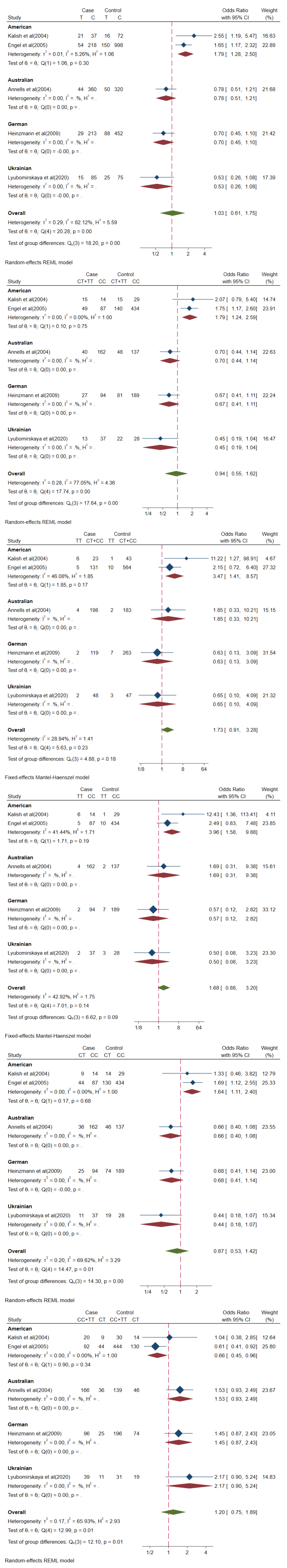

Supplement: Supplementary file 2 [file Image_2.tif]

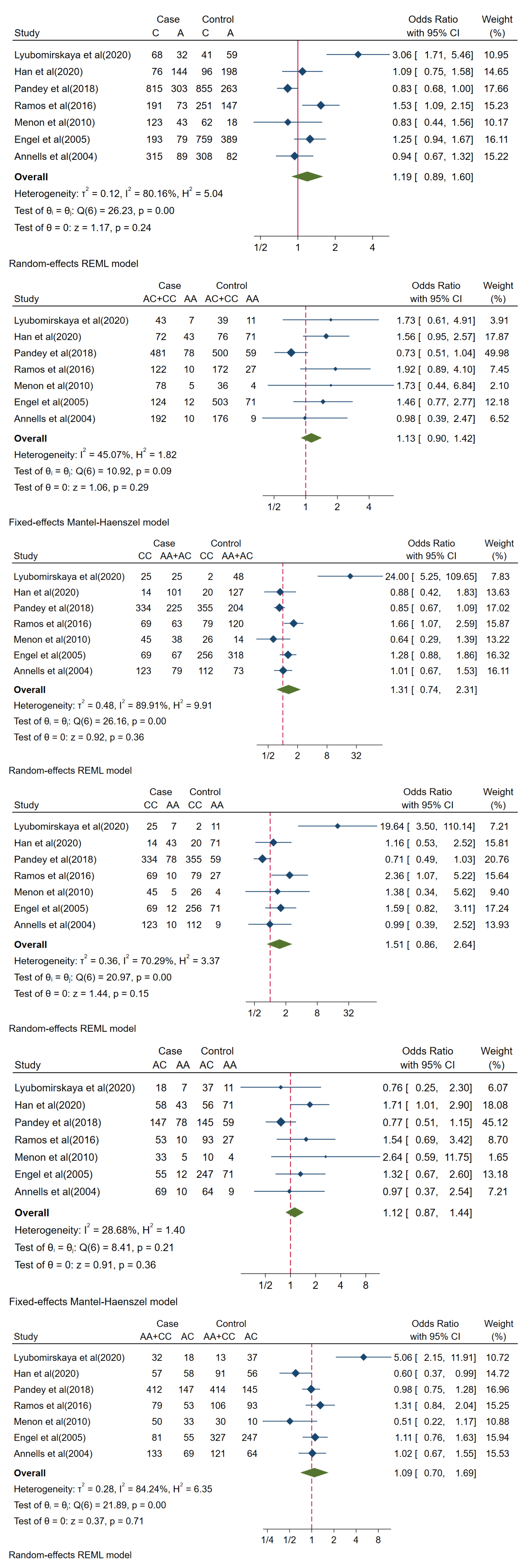

Supplement: Supplementary file 3 [file Image_3.tif]

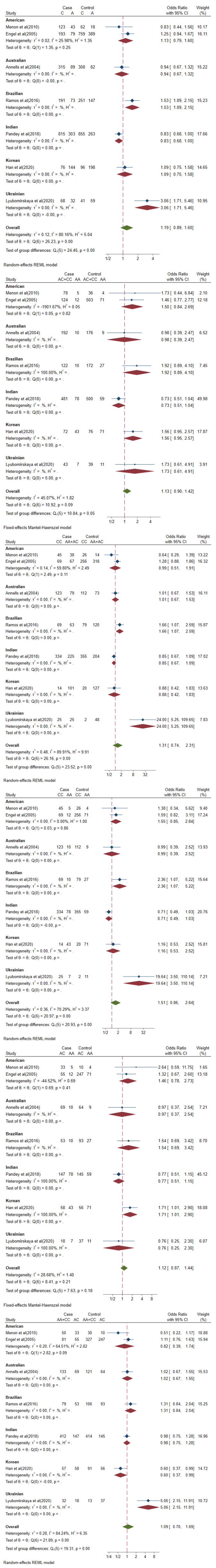

Supplement: Supplementary file 4 [file Image_4.tif]

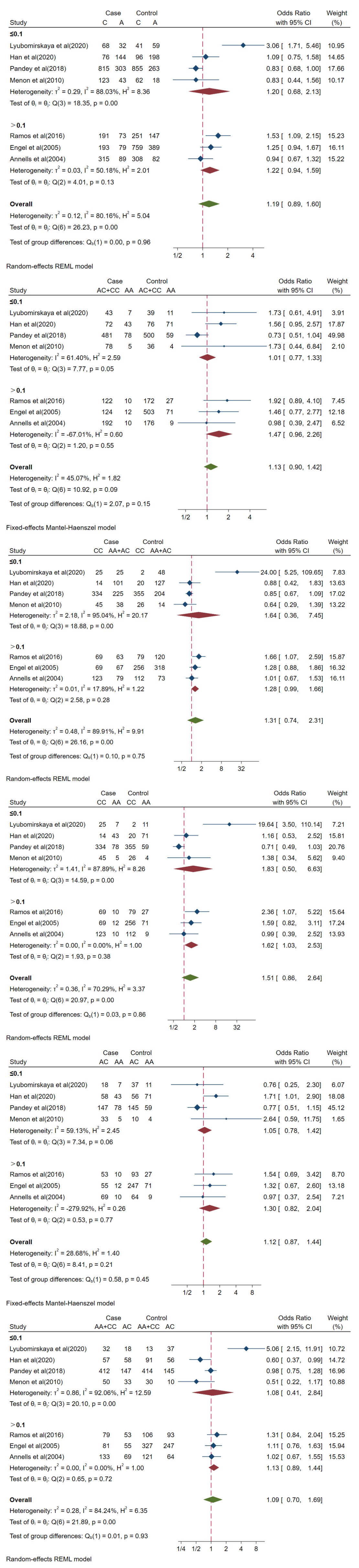

Supplement: Supplementary file 5 [file Image_5.tif]

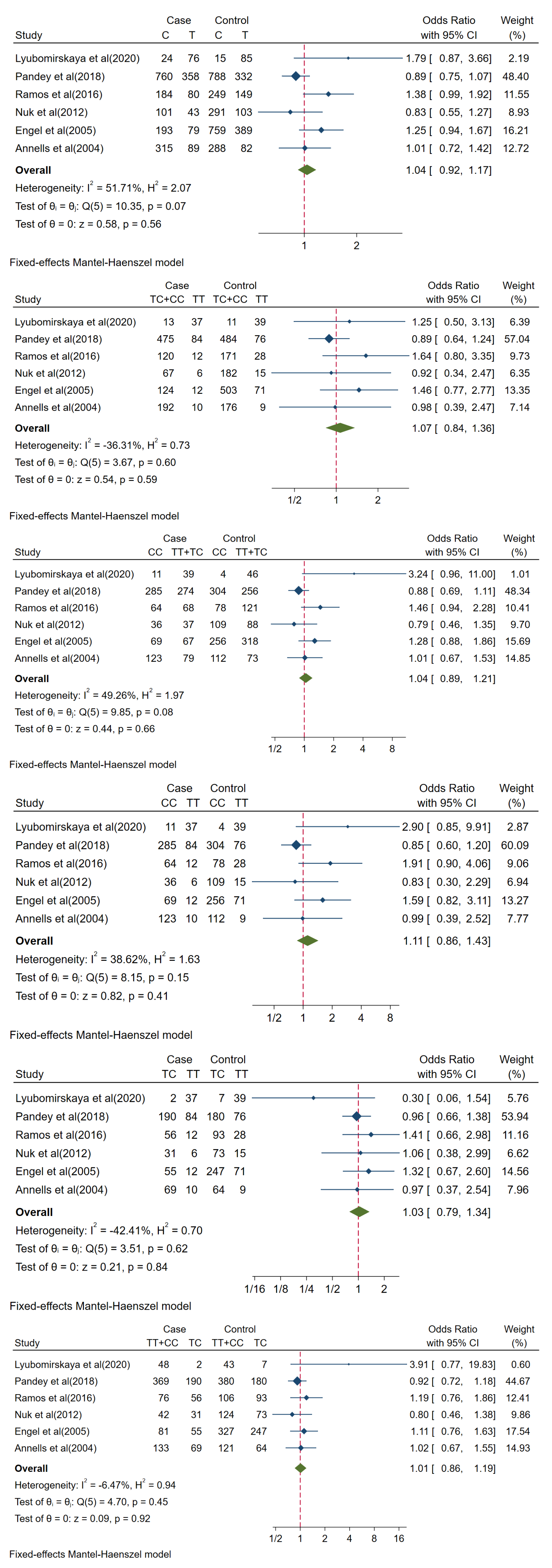

Supplement: Supplementary file 6 [file Image_6.tif]

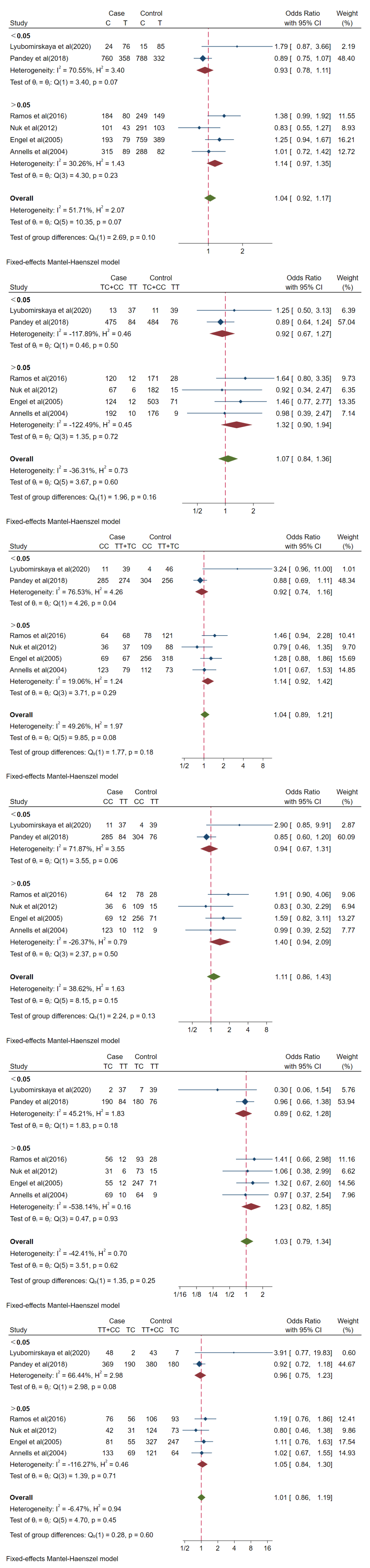

Supplement: Supplementary file 7 [file Image_7.tif]

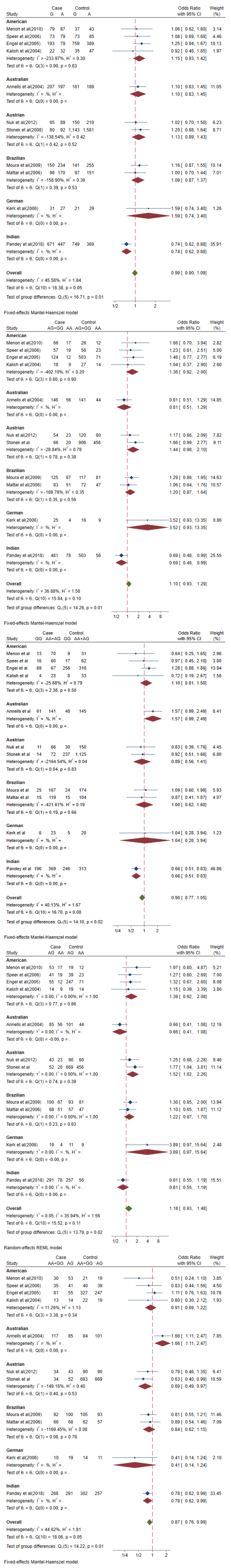

Supplement: Supplementary file 8 [file Image_8.tif]

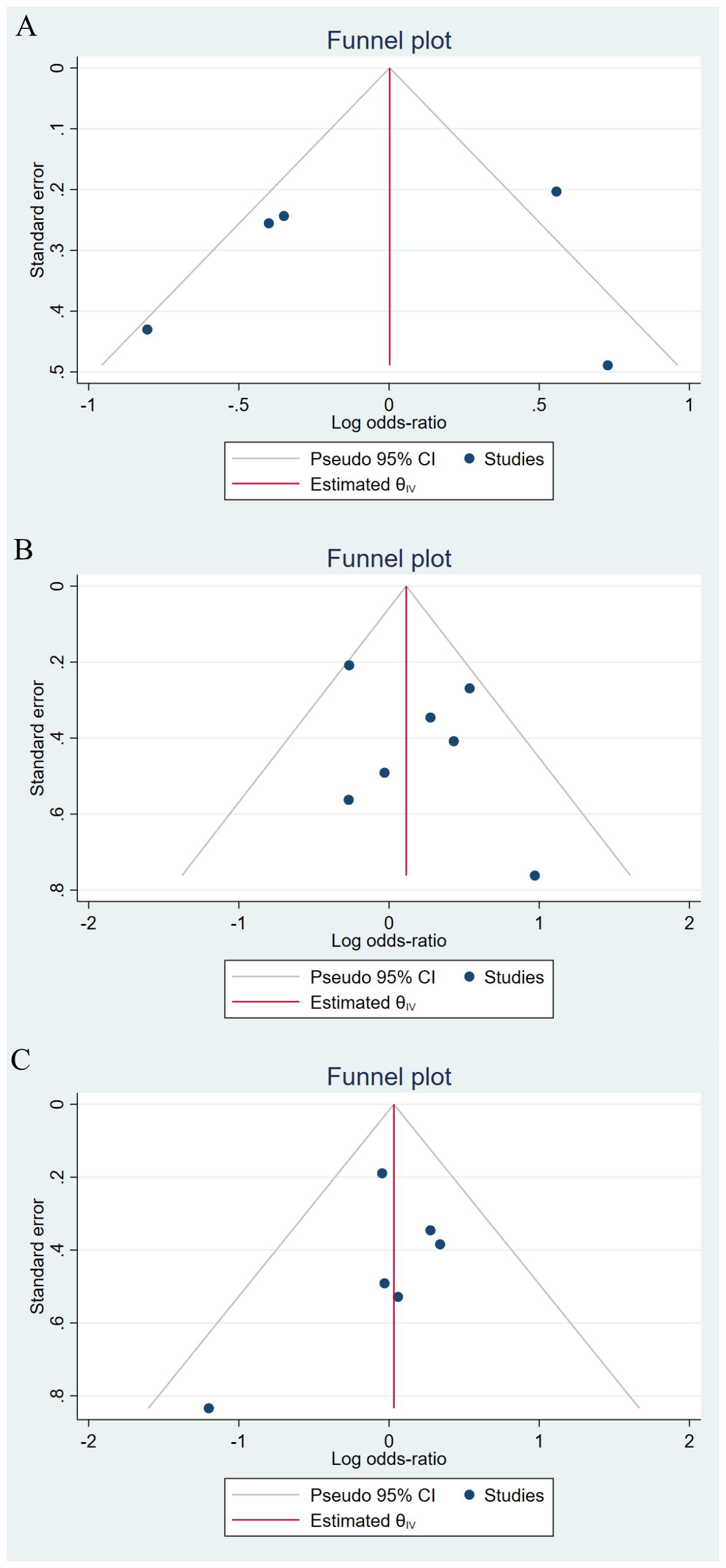

Supplement: Supplementary file 9 [file Image_9.tif]

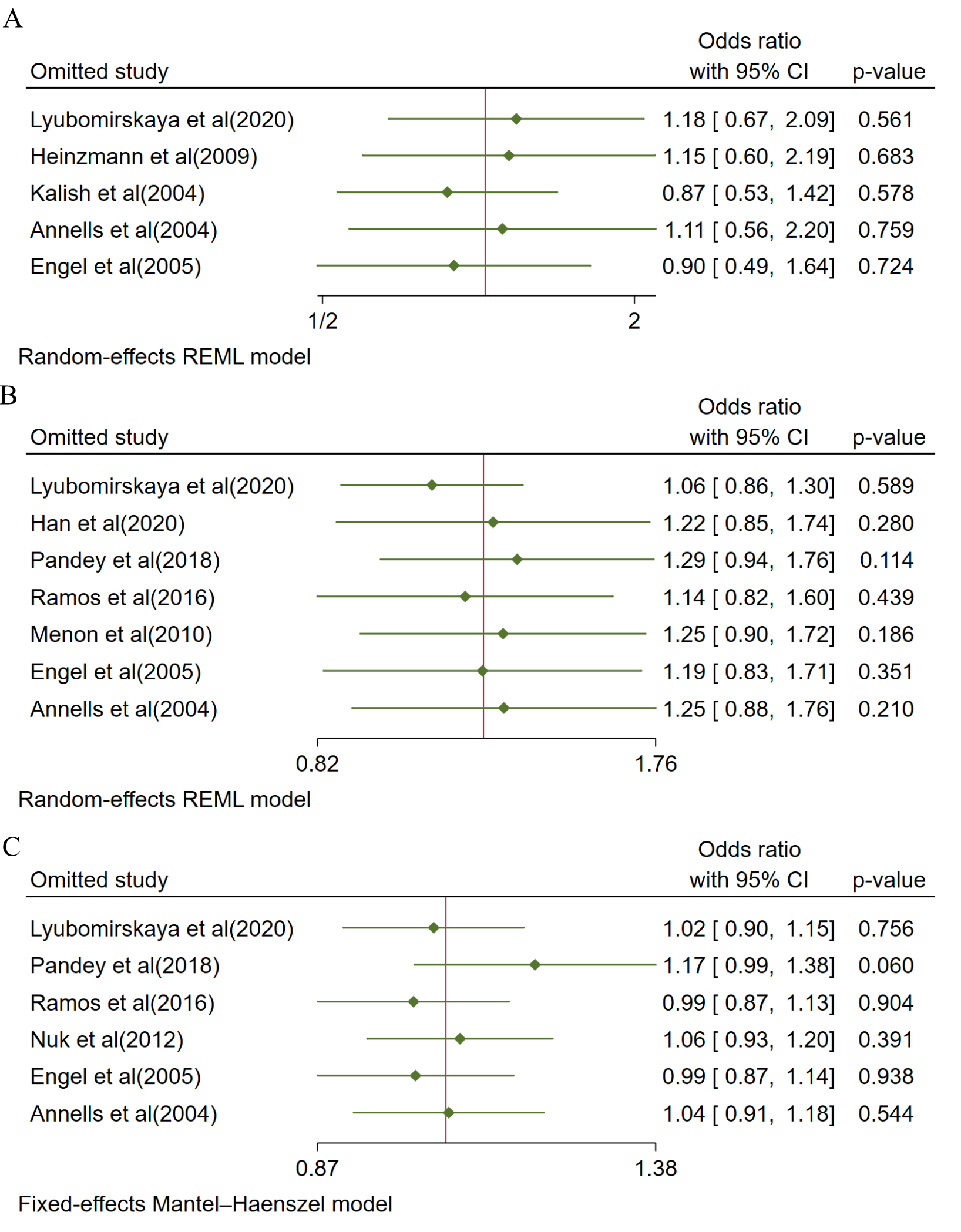

Supplement: Supplementary file 10 [file Image_10.tiff]

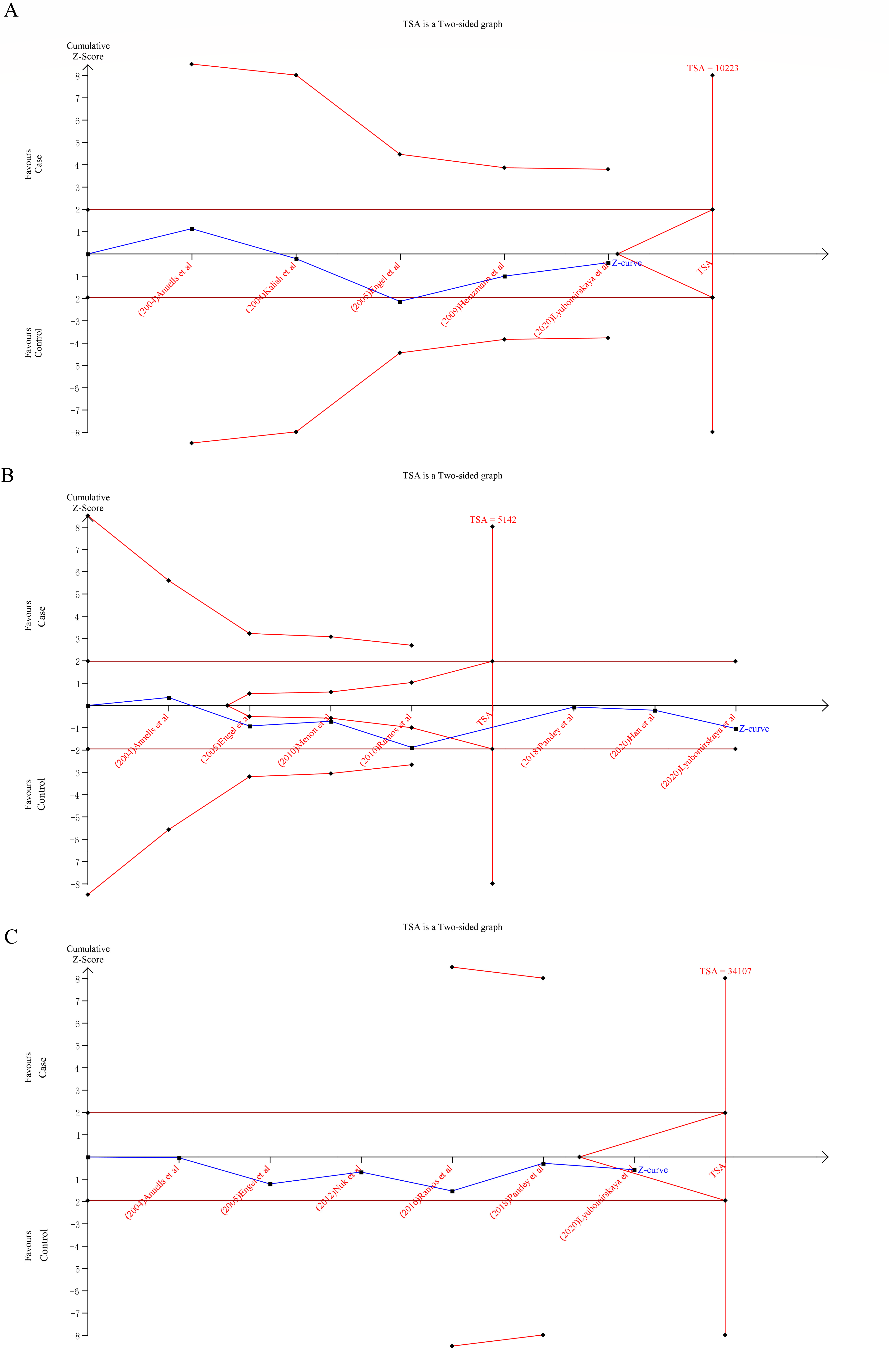

Supplement: Supplementary file 11 [file Image_11.tiff]
