# Supplementary material for: Association of IL-4 and IL-10 Polymorphisms With Preterm Birth Susceptibility: A Systematic Review and Meta-Analysis
Source: Front Immunol. 2022 Jul 4;13:917383. doi: 10.3389/fimmu.2022.917383 (PMC9289468; doi:10.3389/fimmu.2022.917383)
Supplement: Supplementary file 13 [file Table_2.docx]

| **Table S2. eQTL data for SNPs association in Blood** | | | | | |
| --- | --- | --- | --- | --- | --- |
| Chr | SNP | Gene | Tissue | Effective Allele | *P* value |
| 5 | rs2243250 | IL-4 | Blood | T | 6.2×10^-54^ |
| 1 | rs1800872 | IL-10 | Blood | G (or C ) | 4.5×10^-9^ |
| 1 | rs1800871 | IL-10 | Blood | A (or T) | 5.8×10^-91^ |
| 1 | rs1800896 | IL-10 | Blood | C (or G) | 3.7×10^-26^ |
| Chr,chromosome; eQTL,expression quantitative trait Loci. Effective allele in “QTLbase” and the corresponding allele in our article. | | | | | |
